# *Borrelia burgdorferi* Surface Exposed GroEL Is a Multifunctional Protein

**DOI:** 10.3390/pathogens10020226

**Published:** 2021-02-18

**Authors:** Thomas Cafiero, Alvaro Toledo

**Affiliations:** Department of Entomology, Rutgers University, New Brunswick, NJ 08901, USA; tcafiero@princeton.edu

**Keywords:** *Borrelia burgdorferi*, Lyme disease, GroEL, Moonlight protein

## Abstract

The spirochete, *Borrelia burgdorferi*, has a large number of membrane proteins involved in a complex life cycle, that includes a tick vector and a vertebrate host. Some of these proteins also serve different roles in infection and dissemination of the spirochete in the mammalian host. In this spirochete, a number of proteins have been associated with binding to plasminogen or components of the extracellular matrix, which is important for tissue colonization and dissemination. GroEL is a cytoplasmic chaperone protein that has previously been associated with the outer membrane of *Borrelia.* A His-tag purified *B. burgdorferi* GroEL was used to generate a polyclonal rabbit antibody showing that GroEL also localizes in the outer membrane and is surface exposed. GroEL binds plasminogen in a lysine dependent manner. GroEL may be part of the protein repertoire that *Borrelia* successfully uses to establish infection and disseminate in the host. Importantly, this chaperone is readily recognized by sera from experimentally infected mice and rabbits. In summary, GroEL is an immunogenic protein that in addition to its chaperon role it may contribute to pathogenesis of the spirochete by binding to plasminogen and components of the extra cellular matrix.

## 1. Introduction

The spirochete *Borrelia burgdorferi* is the causative agent of Lyme disease [1,2]. The spirochete is transmitted by *Ixodes* ticks and initially infects the host at the site of the tick bite. The spirochete disseminates and invades distant tissues with the help, at least in part, of plasminogen (PLG) [3,4], a zymogen and precursor of plasmin, which is a potent serine protease that degrades plasma proteins. Many outer surface proteins in *Borrelia* can bind to PLG, including OspA [5], OspC [6,7], BBA70 [8], and Erps [9], among others, coating the spirochete with the proenzyme. Subsequently, the urokinase-type plasminogen activator (uPA), a serine protease, converts PLG to plasmin by cleaving the Arg-Val bond in PLG, facilitating the degradation of extracellular matrixes (ECM) and membranes, and enhancing *Borrelia* dissemination in the host [10,11,12]. The active enzyme consists of five kringle domains, each with three disulfide bonds that contain the lysine binding sites and the catalytic domain [13].

Colonization of host tissues is vital for extracellular bacteria. During early infection, *B. burgdorferi* expresses an array of proteins involved in adhesion to host cells [14]. These adhesins bind to different ECM components, facilitating tissue colonization. *Borrelia* has many proteins involved in binding to ECM components, many of which have redundant activities [14,15]. *Borrelia* adhesins bind to laminin, fibronectin, glycosaminoglycans, collagen, and integrins [14]. Interestingly, some adhesins bind to more than one ligand. For example, complement regulator-acquiring surface proteins CspA and CspZ bind to collagen, laminin and fibronectin [16] while BBK32 binds to glycosaminoglycans and fibronectin [17,18]. On the other hand, other proteins seem to bind ligands in a more specific manner. For example, lipoprotein BBA33 binds to collagen type VI [19], whereas Bpg binds to glycosaminoglycan [20].

Canonical outer surface proteins are not the only ones shown to play a role in colonization, dissemination and immune evasion. There are moonlighting proteins in *B. burgdorferi,* such as enolase and HtrA, that are present in multiple subcellular compartments [21,22,23,24,25] and play a role in infectivity [26], persistence [27], PLG [21,22,23], and aggrecan [28] binding. The presence of moonlighting proteins is not rare among prokaryotes. For example, *Streptococcus* species also have glycolytic enzymes with PLG binding properties [29,30,31], including enolase [30], phosphoglycerate kinase [31], and glyceraldehyde-3-phosphate-dehydrogenase [29]. These proteins can be involved in other biological processes, including bacterial adherence, inhibition of complement, and binding to C4b binding protein, among others [31,32,33]. GroEL is an essential cytoplasmic protein detected on the surface of *Bacillus anthracix,* and *Mycoplasma pneumoniae* [34,35]. GroEL binds to host proteins including PLG and components of the ECM [34,35]. The mechanisms by which these proteins are transported remain elusive since they lack membrane-spanning domains, leader sequences or cell wall anchors.

*Borrelia* GroEL (BbGroEL) was detected in different studies associated with the outer membrane (OM) [24,36,37] and outer membrane vesicles (OMV) [21]. The protein is associated with lipid rafts in the OM, but whether it is a surface-exposed protein or not was not determined [37,38]. This study presents evidence that BbGroEL is associated with the OM and is surface exposed. In addition, we show that BbGroEL binds to different host cell proteins, including PLG and ECM components, likely contributing to the dissemination and colonization of host tissues.

## 2. Results

### 2.1. Purification of Recombinant BbGroEL from Escherichia coli Strain Rosetta (DE3)

**Figure 1:**
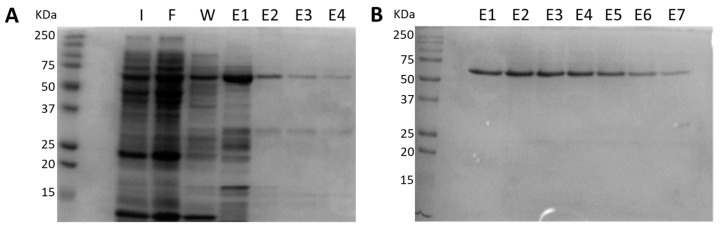
Purification of recombinant BbGroEL. (**A**) Purification of recombinant BbGroEL protein using affinity chromatography; I: Induced *E. coli* (0.3 mM IPTG); F: Flowthrough fraction; W: Wash fraction; E1–E4: Eluted fractions 1–4. (**B**) Eluted fractions were further purified using size exclusion chromatography; E1–E7: Size exclusion eluted fractions.

Recombinant BbGroEL was purified in a two-step process using affinity chromatography and size exclusion. In the first step, the supernatant of an induced *E. coli* Rosetta (DE3) sonicate was subjected to affinity chromatography (Figure 1A). Most of the recombinant BbGroEL was found in the first elution fraction (Figure 1A) with subsequent fractions having modest quantities of the protein. All fractions carried over other *E. coli* proteins. Thus, E1–E4 fractions were pooled together and subjected to size exclusion chromatography (Figure 1B). The elution fractions containing the recombinant BbGroEL did not show contaminants (Figure 1B). Elute fractions were pooled together and used for rabbit immunization and ELISA experiments.

### 2.2. Generation of a Polyclonal Anti-BbGroEL Antibody

**Figure 2:**
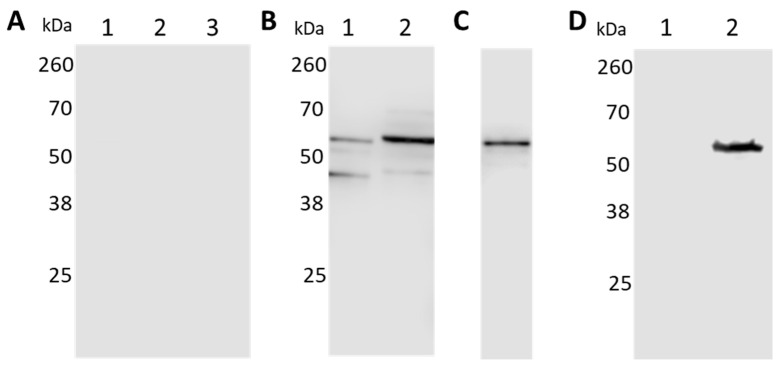
Reactivity of anti-BbGroEL antibody. (**A**) Reactivity of pre-immunized rabbit serum against *B. burgdorferi* (lane 1); *E. coli* (lane 2) and purified GroEL (lane 3). (**B**) Reactivity of rabbit anti-GroEL serum against *B. burgdorferi* (lane 1) and induced *E. coli* Rosetta expressing BbGroEL (lane 2). (**C**) Reactivity of rabbit anti-BbGroEL serum against recombinant BbGroEL. (**D**) Anti-BbGroEL antibody does not recognized *E. coli* GroEL. *E. coli* (lane 1), purified BbGroEL (lane 2).

The purified recombinant BbGroEL protein was used to raise a polyclonal antibody in a New Zealand White rabbit. The reactivity of rabbit serum against recombinant BbGroEL was measured by Western blot analysis. Pre-immunization rabbit serum was used to probe a membrane containing lysates from whole *B. burgdorferi*, *E. coli* Rosetta expressing recombinant BbGroEL, and purified recombinant BbGroEL, none of which reacted with the pre-immunized serum (Figure 2A). In contrast, polyclonal anti-BbGroEL rabbit serum recognized BbGroEL and *E. coli* strain Rosetta lysates expressing BbGroEL, as well as the purified recombinant BbGroEL protein (Figure 2B,C). In addition, the anti-BbGroEL rabbit serum specifically recognized BbGroEL and did not cross-react with *E. coli* GroEL (Figure 2D).

### 2.3. BbGroEL Is Surface Exposed in the OM of B. burgdorferi

**Figure 3:**
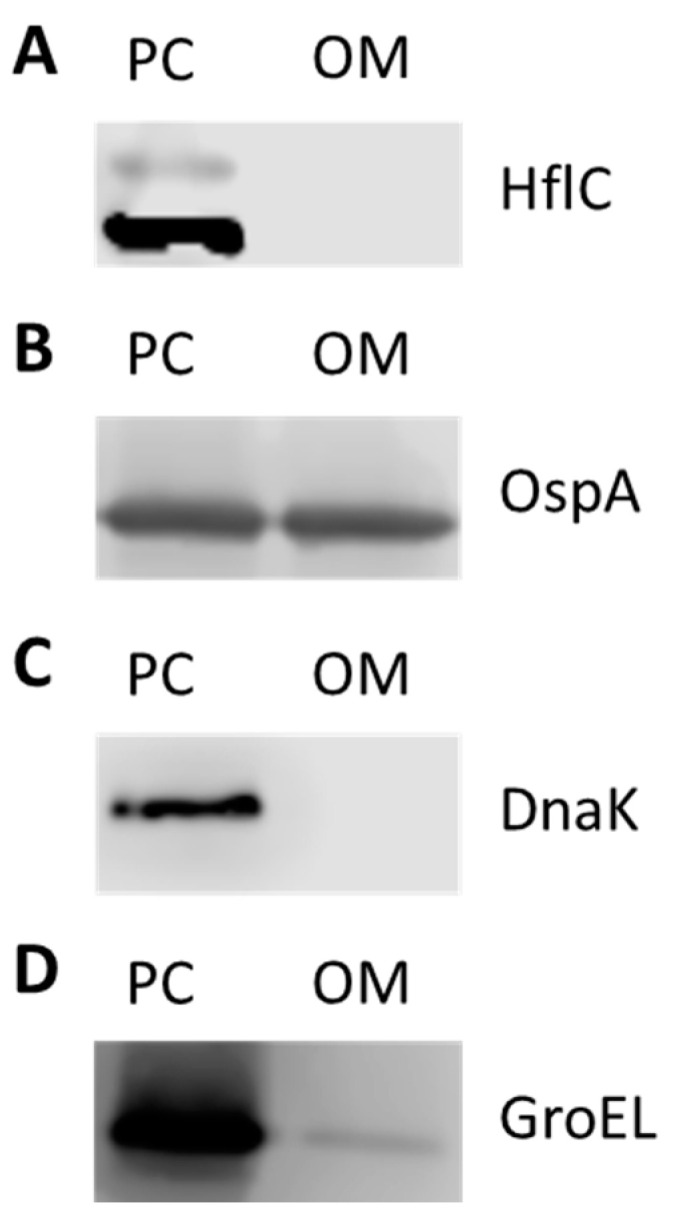
Separation of the protoplasmic cylinder (PC) and outer membrane (OM) fractions. Both fractions were probed with antibodies to detect the inner membrane marker HflC (**A**); the OM protein OspA (**B**); the cytoplasmatic chaperone DnaK (**C**); and BbGroEL (**D**).

**Figure 4:**
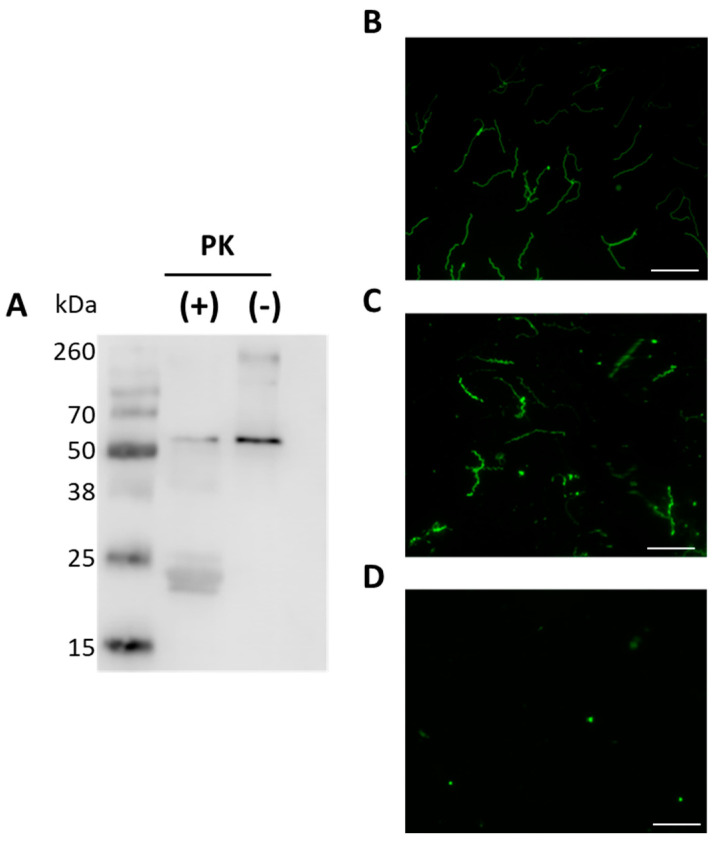
BbGroEL is surface exposed in the OM of *B. burgdorferi*. (**A**) Isolated OM fractions were incubated in the presence and absence of proteinase K and probed with rabbit anti-GroEL. Spirochetes incubated with rabbit anti-BbGroEL antibody (**B**), mouse anti-OspA antibody (**C**) and pre-immune rabbit serum (**D**) were subjected to fluorescence microscope. All scale bars represent 20 µm.

Previous studies showed that BbGroEL is present in OMVs of Borrelia [21] and in the OM of Borrelia [24,37] where it has been associated with lipid rafts [37,38]. The separation and purification of protoplasmic cylinder (PC) and OM fractions of Borrelia was confirmed by Western blot using mouse monoclonal anti-DnaK, rabbit polyclonal anti-HflC and mouse monoclonal anti-OspA antibodies as cytoplasm, inner membrane (IM) and OM markers, respectively (Figure 3A–C). BbGroEL was detected by Western blot in both, PC and OM, fractions (Figure 3D) as noted in previous studies [24,37]. In addition, whether BbGroEL was surface exposed in the OM or not was assessed using two different approaches, including proteinase K digestion of the OM fraction and immunofluorescence of live spirochetes. Proteinase K digestion of the OM fraction followed by Western blot showed almost complete degradation of BbGroEL compared to the OM fraction that was not treated (Figure 4A), which supports that BbGroEL is accessible to proteinase K. In addition, immunofluorescence on live spirochetes were carried out using rabbit polyclonal anti-BbGroEL antibody (Figure 4B), mouse monoclonal anti-OspA antibody (Figure 4C) and pre-immune serum (Figure 4D). As expected, spirochetes incubated with the mouse monoclonal anti-OspA antibody that recognizes the lipoprotein OspA were stained in green (Figure 4C). Similarly, spirochetes incubated with the rabbit polyclonal anti-BbGroEL antibody also showed fluorescence, supporting that BbGroEL is in the OM and is surface exposed.

### 2.4. BbGroEL Binds PLG, and ECM Proteins in a Dose-Dependent Manner

**Figure 5:**
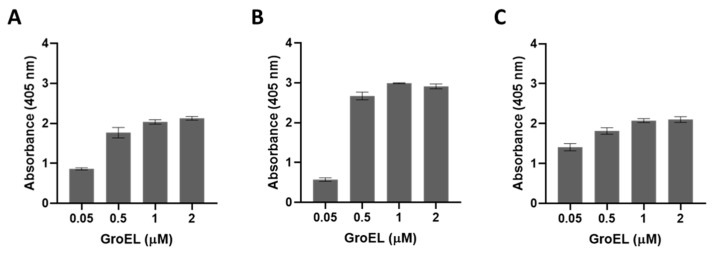
BbGroEL binds to multiple host proteins in a concentration dependent manner. Laminin (**A**), Fibronectin (**B**), and PLG (**C**).

**Figure 6:**
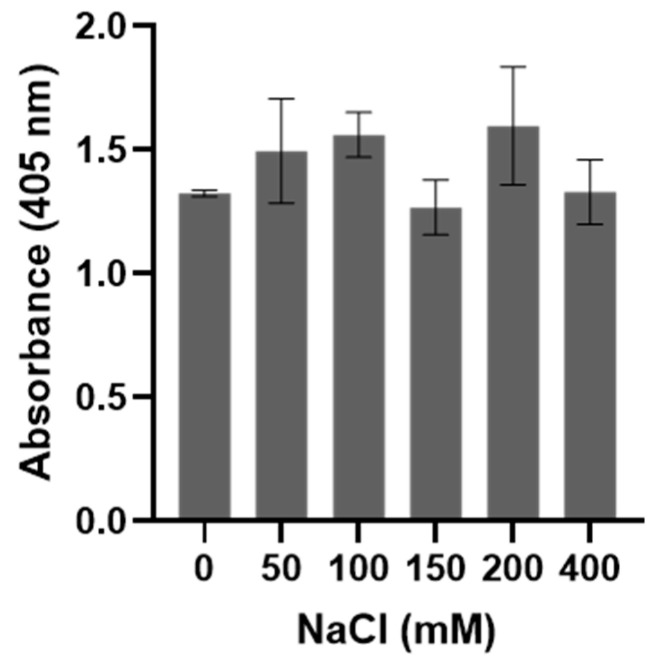
BbGroEL binding to PLG is not mediated by ionic interactions. An ELISA plate was coated with 10 μg/mL of PLG followed by 10 μg/mL of BbGroEL containing NaCl (0–400 mM). Bound BbGroEL was detected using a rabbit anti-BbGroEL antibody.

**Figure 7:**
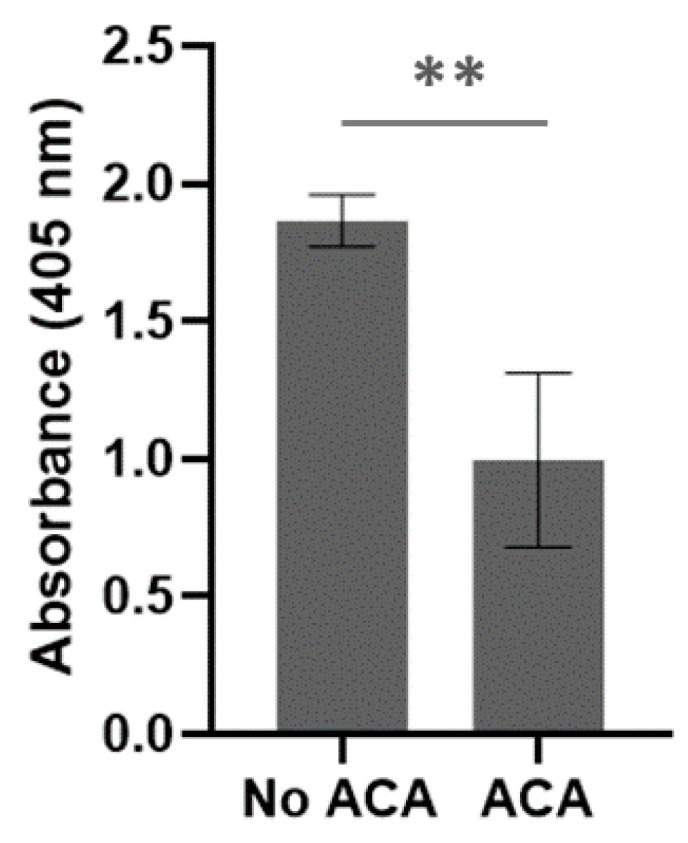
BbGroEL binding to PLG partially depends on Lysine residues. ELISA plates were coated with 10 μg/mL of BbGroEL followed by the addition of 10 μg/mL of human PLG with and without ɛ-aminocaproic acid. Bound PLG was detected using a mouse anti-human PLG antibody. **, *p* ≤ 0.01 (*t*-test).

The presence of BbGroEL in the surface of the OM and the OMVs of the spirochete suggests that it is a moonlighting protein that could interact with different host’s proteins. Thus, we tested whether BbGroEL was an adhesin or could be involved in dissemination by immobilizing PLG, fibronectin and laminin onto ELISA plates and incubating these proteins with increasing concentrations of BbGroEL (0.05–2 µM). The results showed that the binding of BbGroEL to PLG, laminin and fibronectin was dose dependent (Figure 5) and was not inhibited by the addition of increasing concentration of NaCl (Figure 6), which supports that binding is not mediated by ionic interactions. On the other hand, the binding of BbGroEL to PLG was significantly decreased (*p* < 0.01) by the addition of ε-aminocaproic acid (Figure 7), a well-known lysine analog. This result supports the role of lysine residues in the interaction between BbGroEL and PLG.

### 2.5. BbGroEL Is an Immunogenic Protein

**Figure 8:**
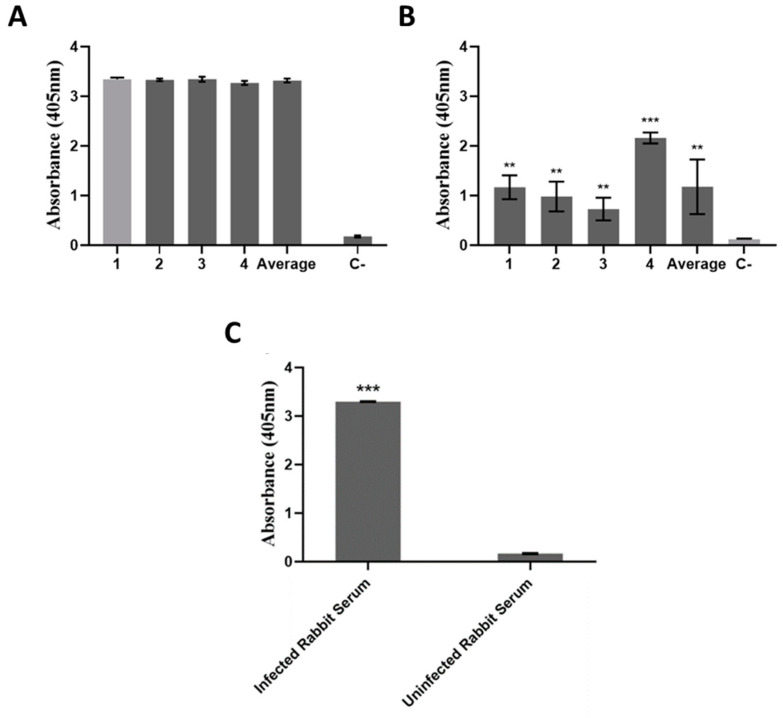
BbGroEL is an immunogenic protein. (**A**) Serological reactivity of experimentally infected mice (1:100) to *B. burgdorferi* whole cell extracts. (**B**) Serological reactivity of experimentally infected mice (1:100) to recombinant BbGroEL. (**C**) Serological reactivity of *B. burgdorferi* tick infected rabbit (1:100) to recombinant BbGroEL. **, *p* ≤ 0.01; ***, *p* ≤ 0.001.

To test the immunogenicity of BbGroEL, sera from mice and a rabbit infected with *B. burgdorferi* were tested against Borrelia whole-cell lysates and purified BbGroEL (Figure 8A,B). Sera from needle-infected mice recognized Borrelia lysates, and their responses were consistent and similar (Figure 8A). The recombinant BbGroEL was also recognized by sera from needle-infected mice (Figure 8B) and a tick-infected rabbit (Figure 8C). The response in all cases was significantly higher than the one observed in their respective controls (non-infected mouse serum and pre-infection serum from the rabbit). Together, these results support that BbGroEL contributes to trigger an immune response during the course of the spirochetal infection.

### 2.6. BbGroEL Protein Production

**Figure 9:**
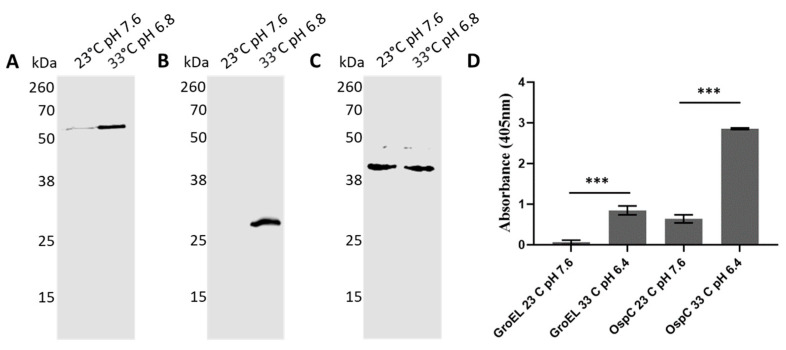
BbGroEL protein production increases under conditions that mimic the mammalian host. (**A**–**C**) Western Blot showing the expression of BbGroEL (**A**) OspC (**B**) and FlaB (**C**) at 23 °C pH 7.6 and 33 °C pH 6.8. (**D**) ELISA results showing the differential expression of BbGroEL and OspC at 33 °C pH 6.8 and 23 °C pH 7.6. ***, *p* ≤ 0.001 (*t*-test).

The production of *B. burgdorferi* proteins is tightly regulated throughout its enzootic cycle, where it alternates between the tick vector and the vertebrate host. While transitioning from the tick to the vertebrate milieu, the spirochetes experience dramatic changes in protein expression. A notable example of a switch in protein expression is OspC, which is required to establish the infection in the mammalian host [39,40,41,42]. These changes in the milieu can be mimicked in culture, at least to some extent, by changing the temperature and pH. To test whether these switches affected BbGroEL protein production, spirochetes were grown at 23 °C pH 7.6 and 33 °C pH 6.8, to mimic tick and mammalian conditions, respectively. Protein production under both conditions was compared by Western Blot and ELISA. Western blot results showed that there are increased levels of BbGroEL and OspC at 33 °C pH 6.8 compared to 23 °C pH 7.6 (Figure 9A,B) while there are no changes in the levels of FlaB (Figure 9C). These results were further confirmed by ELISA, which showed a significant increase in BbGroEL and OspC protein levels at 33 °C pH 6.8 compared to 23 °C pH 7.6.

## 3. Discussion

*B. burgdorferi* infection is facilitated by binding to some host proteins, including components of the host’s ECM and PLG [14,43]. The spirochete uses an array of OM proteins for binding, some of which cope with multiple functions by interacting with different host proteins through non-overlapping domains [43]. Nonetheless, canonical OM proteins are not the only proteins that interact with the host’s proteins. There is increasing evidence that moonlighting proteins in *B. burgdorferi*, as well as in other bacteria, including *Streptococcus* spp., *Staphylococcus* spp., *Mycoplasma* spp., *Clostridium* spp., *Salmonella* spp., and *Helicobacter* spp., play a critical role in tissue colonization and immune evasion [21,22,23,29,30,33,44,45,46,47]. Despite lacking signal peptides, many moonlighting proteins are associated with the OM and OMV [48]. *Borrelia*, like gram-negative bacteria, shed OMV [49,50] that are known to be involved in pathogenic processes of Lyme borreliae [51]. *Borrelia*’s OMV include multiprotein complexes of OM proteins [52], moonlighting proteins such as HtrA and enolase [21], as well as adhesins, that mediate in binding to host cells [50]. The presence of proteins with multiple functions and redundant properties is common in *B. burgdorferi* [43] and are responsible for tissue adhesion and colonization [14,43]. The redundancy of functions that many proteins show in *B. burgdorferi* highlights the importance of adhesion for tissue colonization. In addition, the presence of proteins that cope with multiple functions confers adaptability to an organism with a small genome like *B. burgdorferi*.

In this study, we looked into the moonlighting properties of BbGroEL, a canonical cytoplasmic chaperone protein that is responsible for the folding of newly synthesized proteins into their functional counterparts by sequestering nonnative polypeptides and forming an aggregated functional protein [53]. BbGroEL has been found associated with the OM and OMVs [21,24,36,37]. The method of choice for analyzing whether a protein is surface expose or not is a PK treatment of whole cells. Nonetheless, GroEL is much more abundant in the cytoplasm than in the outer membrane, which limits the applicability of PK treatment in whole cells. In this study, we used two independent and complementary approaches to demonstrate that the protein is surface expose, an IFA and proteinase K treatment of the OM. Combined, the results obtained by PK treatment of the OM and IFA of whole cells suggest that BbGroEL is surface exposed, which supports that BbGroEL interacts with host’s proteins. Furthermore, we showed that BbGroEL acts as an adhesin by binding to components of the ECM. This is not surprising since GroEL also functions as an adhesin in *Lactobacillus johnsonii* [54], *Clostridium difficile* [55], *Salmonella enterica* serotype Typhimurium [56], *Listeria monocytogenes* [57], and *Helicobacter pylori* [58,59]. In fact, specific antibodies to GroEL can alter the ability of *C. difficile* to bind and colonize the intestine of vaccinated mice [60].

There are a large number of proteins associated with the PLG system in *Borrelia* [3]. Both relapsing fever and Lyme disease *Borrelia* species bind and fix PLG onto their membranes [3,61,62]. Subsequently, PLG is activated into plasmin, acting as a bound protease and facilitating the dissemination of the spirochete in the host by degrading the ECM [3,61,63]. In addition, *B. burgdorferi* induces the synthesis of the urokinase PLG activator (uPA) and its receptor, uPAR, that can facilitate the dissemination of the spirochete by activating the membrane-bound PLG [10,11,12,64,65,66]. The proenzyme PLG has five kringle domains that contain the lysine binding sites and the catabolic domain. Thus, the presence of lysine residues in PLG binding proteins is critical. BbGroE has 543 amino acids, 53 of which are Lys. The addition of ε-aminocaproic acid (ACA), a structural analog of lysine, competes with PLG-binding for Lys binding sites present in the kringle domains, which is consistent with the results we obtained for BbGroEL. In contrast, ionic interactions do not mediate in the binding of this protein to PLG since increasing concentrations of NaCl had no impact on the results. These results support the role of BbGroEL as a PLG-binding protein. The role of this chaperone protein in binding to PLG is a trait present in other bacteria. For example, *Mycoplasma pneumoniae* has a surface exposed GroEL protein that binds to PLG [34]. Although PLG is better known for its potent proteolytic activity that can facilitate bacterial dissemination, it also serves as a co-factor in adhesion [67]. GroEL is not the only moonlighting protein with PLG binding capabilities. Glycolytic proteins, including enolase [21,22,23,30,32,68], phosphoglycerate kinase [22,31], and glyceraldehyde-3-phosphate-dehydrogenase [29] from different bacteria and protozoa species also bind to PLG.

Lastly, *B. burgdorferi* tightly regulates gene expression while transitioning from the tick vector to the mammalian host. As a result, the protein composition of the OM changes dramatically. The quantity of BbGroEL changed upon temperature and pH shifts that mimic, with limitations, the host environment [39,69,70]. It is important to note that expression of GroEL in other bacteria is controlled by positive and negative mechanisms, or a mix of them [71]. For example, in *Pseudomonas aeruginosa*, *Vibrio cholera*, and *E coli* the expression of *groEl* is controlled positively by RpoH (σ^32^) [72,73,74]. However, *B. burgdorferi* does not have a σ^32^ homolog. Alternatively, other organisms use heat-shock transcriptional regulators that function as repressors, including HrcA, HspR, and CtsR [71], which are absent in *B. burgdorferi.* Interestingly, the quantity of GroEL seems to increased more in the membrane that in the cytoplasm at increasing temperatures [36] but the gene expression does not change with a temperature swift [75]. Collectively, these finding suggest that there is a negative regulator that prevents production of GroEL at lower temperatures.

Interestingly, BbGroEL is recognized by sera from a rabbit and mice infected with *B. burgdorferi* via tick bite and needle, respectively. This result confirms the immunogenicity of BbGroEL, which has also been shown in other spirochetes, including different pathogenic strains of *Leptospira* spp. [76] and *Treponema pallidum* [77]. Nonetheless, GroEL in these spirochetes is not present in the OM [77,78,79] and therefore not involved in binding to PLG or ECM components.

## 4. Materials and Methods

### 4.1. Bacteria, Cultures, and Sera from Laboratory Animals

All *B. burgdorferi* strain B31 cultures were grown to mid-log phase in BSK-II medium supplemented with 6% rabbit serum (Sigma, St. Louis, MO, USA) at 33 °C; except cultures used for the shift protein expression experiment that were grown at 33 °C and pH 6.8 and 23 °C and pH 7.6. *Escherichia coli* DH5α and Rosetta (DE3) (Novagen, Madison, WI, USA) were grown in LB media (Fisher Scientific, Pittsburgh, PA, USA) in the presence of kanamycin (50 µg /mL). A New Zealand White rabbit (Charles River, Wilmington, MA, USA) was inoculated intradermally with 100 μg of recombinant BbGroEL in complete Freund’s adjuvant followed by two boosters of 50 μg of the recombinant BbGroEL in incomplete Freund’s adjuvant. Mouse sera from C3H/HeN mice (Jackson Laboratories, Bar Harbor, ME, USA) infected with 2 × 10^4^ spirochetes were collected 4 weeks after inoculation. *B. burgdorferi* infected rabbit serum was generously provided by Dr. Jorge Benach.

### 4.2. Purification of the OM and PC from B. burgdorferi

The separation of the OM and IM was carried out as previously described [37,80,81]. Briefly, spirochetes were grown in complete BSK-II to late-log phase and harvested by centrifugation at 5800× *g* for 20 min. After washing with PBS containing 0.1% BSA, spirochetes were incubated in a 25 mM citrate buffer (pH 3.2) in agitation for 2 h at room temperature to separate the OM and the PC. Both fractions were subsequently isolated by using a discontinuous sucrose gradient (56, 42, 25% from bottom to top) followed by a continuous sucrose density gradient (10–40%, top to bottom). Lastly, the isolated PC fraction was diluted (1:5) in PBS, pelleted at 10,000× *g* for 20 min, resuspended in PBS and stored at −80 °C. The purified OM fraction was diluted in PBS (1:5), centrifuged at 141,000× *g* for 4 h, and the pellet was resuspended in a 1 mM phenylmethylsulfonyl fluoride (PMSF) solution and stored at −80 °C.

The purity of both fractions was evaluated through the use of antibodies that targeted proteins present in the OM, OspA [82], IM, HflC [37,83], and in the cytoplasm, DnaK [84].

### 4.3. Recombinant Protein Expression and Purification

N-terminal Histidine-tagged GroEL was created in a pET-28a(+) vector (EMD Chemicals Inc., Gibbstown, NJ). Briefly, the GroEL gene (bb0649) was amplified using primers GroELF (5′-ATGGCTAAAGACATATATTTT-3′) and GroELR (5′-TTACATCATTCCCATTCCTGG-3′) followed by digestion with the restriction enzymes Ndel and Xhol (New England BioLabs, Ipswich, MA, USA).

Recombinant BbGroEL was expressed in *E. coli* Rosetta upon induction with 0.3 mM isopropyl-β-d-thiogalactopyranoside (IPTG). Cells were centrifuged at 11,000× *g* at 4 °C for 10 min, and the pellet was washed with PBS (VWR International, Radnor, PA, USA). The pellets were sonicated and the supernatant collected for affinity chromatography.

A 5 mL gravity flow column (G-Biosciences, St. Louis, MO, USA) was prepared with a Ni-Sepharose resin (GE Healthcare, Chicago, IL, USA). The supernatant was run through the column, and the flow-through was collected and stored at 4 °C for sodium dodecyl sulfate-polyacrylamide gel electrophoresis (SDS-PAGE) analysis.

An initial wash step was performed by adding a wash-bind buffer (25 mM Tris-HCl, 300 mM NaCl, 25 mM imidazole, 1 mM DTT) to the column. The wash fraction was collected and stored at 4 °C for SDS-PAGE analysis. Subsequently, the elution buffer (25 mM Tris-HCl, 300 mM NaCl, 250 mM imidazole, 1 mM DTT) was added to the column. Eluted fractions (1 mL) were collected and kept at 4 °C until analysis by SDS-PAGE. Induced cell extract, flow-through, wash, and elute fractions were all assessed by SDS PAGE analysis followed by staining with coomassie brilliant blue (Sigma, St. Louis, MO, USA).

The elute fractions were subjected to gel filtration chromatography. Briefly, Sephadex G-75 media (GE Healthcare, Chicago, IL, USA) was added to a glass chromatography column (Ace Glass Inc., Vineland, NJ, USA) and the eluted fractions from the previous purification were run through. Collected fractions were analyzed by SDS-PAGE.

Samples containing the purified protein were pooled and subjected to a buffer exchange (25 mM Tris HCl, 150 mM NaCl, 5% glycerol) using an Amicon Ultra-15 Centrifugal filter unit (5000× *g*) (Millipore Sigma, St. Louis, MO, USA).

### 4.4. Western Blot Analysis

Different Western blot assays were conducted to analyze the polyclonal anti-BbGroEL antibody activity, separation of OM and PC fractions, and proteinase K digestion of OM. Briefly, 1 µg of purified BbGroEL, 2 µg of purified OM and PC fractions, 1 µg of *E. coli* lysate or 10^7^ spirochetes were resuspended in 20 μL of PBS and received 10 μL of 3 times SDS-PAGE sample buffer containing 2-mercaptoethanol (Millipore Sigma, St. Louis, MO, USA). Samples were boiled for 5 min and loaded into 12.5% SDS-PAGE gels and separated by electrophoresis. Gels were transferred to a nitrocellulose membrane (Millipore Sigma, St. Louis, MO, USA) and blocked for 1 h with 5% non-fat milk powder in TBST. The primary antibodies used for Western blot include rabbit polyclonal anti-HflC [83], monoclonal anti-OspA (mouse IgG1) antibody [85], monoclonal anti-DnaK (mouse IgG1) [85], polyclonal rabbit anti-BbGroEL serum, or mouse anti-FlaB [86]. After incubation with a primary antibody for 1 h, membranes were washed 3 times with TBST and incubated with a secondary antibody, either HRP-conjugated goat anti-Mouse IgG (LI-COR Biosciences, Lincoln, NE, USA) or HRP-conjugated goat anti-Rabbit IgG (Immunoreagents, Raleigh, NC, USA) for 1 h. The membranes were incubated in WesternSure^®^ PREMIUM Chemiluminescent Substrate (LI-COR Biosciences, Lincoln, NE) and visualized by LI-COR C-DiGit Blot Scanner (LI-COR Biosciences, Lincoln, NE). Protein concentration for all samples were calculated using a Bradford protein assay (Thermo Scientific, Waltham, MA, USA), and equal amounts loaded to a SDS-PAGE gel.

### 4.5. ELISA

The 96 well enzyme-linked immunosorbent assay (ELISA) plates (Thermo Scientific, Waltham, MA, USA) were coated overnight at 4 °C with 1 μg of BbGroEL, PLG, fibronectin, laminin, or *B. burgdorferi* lysate in bicarbonate-carbonate coating buffer (50 mM NaCO3, 50 mM NaHCO3). ELISA plates were blocked with 1% casein (Thermo Scientific, Waltham, MA, USA) for 1 h at 37 °C and washed with PBS. The plate was incubated with polyclonal rabbit anti-BbGroEL serum or rabbit anti-human PLG at 37 °C for 1 h and washed 3 times with PBS. Different ELISA experiments were carried out as follows: (i) To detect bound BbGroEL to ECM (laminin, and fibronectin), and PLG rabbit polyclonal anti-BbGroEL was incubated for 1 h at 37 °C, washed 3 times with PBS, and incubated with a goat anti-rabbit IgG-alkaline phosphatase conjugate (Sigma) at 37 °C for 1 h. The experiment was repeated using increasing concentrations of NaCl to address whether PLG binding was mediated by ionic interactions. (ii) To detect PLG bound to BbGroEL in the presence or absence of ε-aminocaproic acid (ACA), rabbit anti-human PLG (Boehringer, Rheim, Germany) was incubated for 1 h at 37 °C, washed 3 times with PBS, and incubated with goat anti-rabbit IgG-alkaline phosphatase conjugate (Sigma) at 37 °C for 1 h. (iii) To test the immunogenicity of BbGroEL, immobilized *B. burgdorferi* and recombinant BbGroEL were incubated with sera from infected mice and a rabbit for 1 h at 37 °C, washed 3 times with PBS, and incubated with goat anti-mouse IgG and goat anti-rabbit IgG-alkaline phosphatase conjugate at 37 °C for 1 h. (iv) To assess protein expression of BbGroEL upon temperature and pH shift, cell extracts from *B. burgdorferi* grown at 33 °C pH 6.8 and 23 °C pH 7.6 and probed with rabbit anti-BbGroEL for 1 h at 37 °C, washed 3 times with PBS, were incubated with goat anti-rabbit IgG-alkaline phosphatase conjugate at 37 °C for 1 h. Lastly, the plate was washed three times with PBS, followed by incubation with alkaline phosphatase substrate (Sigma, St. Louis, MO, USA) for 30 min at room temperature. The absorbance was read at OD_405_ using a Thermo Scientific Multiskan GO microplate Spectrophotometer (Fisher Scientific, Pittsburgh, PA, USA). Casein 1% was used as a negative control to detect unspecific binding and the absorbance values used as a blank. A secondary control was included in all experiments (all the components added except the primary antibody) to assess cross-reactivity of the secondary antibody.

### 4.6. Immunofluorescence Assay

*B. burgdorferi* cells were collected at mid log phase and washed three times with Hank’s balanced salt solution (HBSS) (Gibco Laboratories, Gaithersburg, MD, USA). Spirochetes were incubated in HBSS in the presence of rabbit anti-BbGroEL serum or monoclonal anti-OspA at 33 °C for 1 h. Spirochetes were centrifuged at 10,000× *g* and washed three times with HBSS. Spirochetes were then fixed onto a teflon microscope slide (Carlson Scientific, Peotone, IL, USA) with 100% methanol at −20 °C, dried at 33 °C, and washed with HBSS. Fluorescein isothiocyanate (FITC) conjugated Goat anti-rabbit antibody (Invitrogen, Carlsbad, CA, USA) was added to the wells and incubated at 33 °C in a wet chamber for 1 h. The slide was dried at 33 °C, washed in HBSS, and dried again. Slides were mounted with SlowFade Diamond Antifade Mountant (Thermo Scientific, Waltham, MA) and viewed with a Zeiss ApoTome.2 microscope (ZEISS, Oberkochen, Germany).

### 4.7. PK Treatments

Proteinase K (PK) treatment of OM was done similarly to PK treatments of whole cells as previously described [87,88,89]. Briefly, purified OM fractions were resuspended in 1 mL PBS or PBS with PK (Boehringer) at a concentration of 200 μg/mL. Samples were incubated by agitation for 1 h at room temperature (23 °C). A protease inhibitor cocktail (EDTA-free; Roche Diagnostics, Indianapolis, IN, USA) was used to stop protein digestion. Samples were centrifuged and washed with PBS. Samples were then subjected to SDS-PAGE and Western blot as previously described.

### 4.8. Effect of Temperature and pH Shift on the Protein Expression of BbGroEL

*B. burgdorferi* was grown at 35 °C until it reached the midlog phase. This culture was diluted to 10^6^ bacteria/mL and grown at 23 °C pH 7.6 until it reached a density of 1 × 10^7^ bacteria/mL. The culture was diluted to a density of 2.5 × 10^5^ bacteria/mL and separate cultures were incubated at 23 °C pH 7.6 and at 35 °C pH 6.8 until they reached a mid-exponential phase. Cultures at the same density were lysed using BugBuster protein extraction reagent (Millipore Sigma, St. Louis, MO, USA) and subsequently tested by Western blot and ELISA as previously described.

### 4.9. Statistics

Data was analyzed on GraphPad Prism 8.3.0 (GraphPad Software Inc., San Diego, CA, USA) using unpaired *t*-tests to compare two groups or ANOVA to compare multiple groups.

## Data Availability

The data presented in this study are available upon request.
